# Biophysical Modeling of Lipopolysaccharides in Gram-Negative Bacteria: From Atomic to Colloidal Models

**DOI:** 10.3390/ijms27052488

**Published:** 2026-03-08

**Authors:** Alexander N. Shvirst, Timur V. Mamedov, Andrey A. Butanaev, Alexander G. Pogorelov, Gennady N. Chuev

**Affiliations:** Institute of Theoretical and Experimental Biophysics, Russian Academy of Sciences, 142290 Puschino, Russia; shvirst93@bk.ru (A.N.S.); attore7777777@gmail.com (T.V.M.); agpogorelov@rambler.ru (A.G.P.)

**Keywords:** lipopolysaccharides, O-antigen, bacterial outer membrane, bilayer, molecular model, coarse-grained simulations, physicochemical properties, polymer brush, colloidal model

## Abstract

Lipopolysaccharides (LPSs) are key components of the bacterial outer envelope, determining its structural integrity, barrier properties, and interactions with the surrounding environment. This review analyzes the relationship between the molecular architecture of LPSs and their physicochemical properties. Particular attention is being paid to the organization of LPS-containing supramolecular assemblies, including bacterial outer membranes, bilayers, micelles, and LPS brushes. The review further focuses on theoretical frameworks employed to describe LPS layers and discusses the physical meaning of the parameters involved in these models. The simulations involve a wide range of approaches starting from all-atom molecular treatment and up to polymer and colloidal approaches. When considering these models, we focus on the relationships between parameters that are addressed at each level of modeling. It is shown that biological functions such as membrane stability and bacterial adhesion are largely governed by the molecular organization of LPS. This structure–property relationship provides a basis for predicting the performance of anti-adhesive biomaterials, antimicrobial strategies, and bactericidal agents.

## 1. Introduction

Lipopolysaccharides (LPSs) are the dominant components of the outer membrane of Gram-negative bacteria and represent a unique class of biological macromolecules that play a central role in microbiology and immunology [1,2]. These structures not only constitute the primary structural element responsible for the integrity and barrier function of the bacterial envelope but also act as potent immunostimulatory agents that critically contribute to bacterial pathogenicity and to the initial stages of bacterial adhesion to a wide variety of surfaces [3].

The complexity and variability of LPS molecular organization, from the hydrophobic lipid A moiety through the conserved oligosaccharide core to the highly polymorphic O-antigen, directly determine their diverse physicochemical properties and, consequently, their biological functions. Understanding the relationship between the hierarchical structure of LPSs and their fundamental properties, such as amphiphilicity, electrostatic characteristics, conformational flexibility, and their ability to form supramolecular assemblies, is, therefore, of critical importance. This knowledge underpins a broad range of applications, including the development of novel antimicrobial strategies aimed at overcoming the outer membrane barrier of bacteria, the design of biocompatible materials for controlling bacterial adhesion, and the elucidation of mechanisms underlying the innate immune response to endotoxins [2,3,4].

This review provides a comprehensive analysis of biophysical models of LPSs and the key physicochemical properties that govern LPS behavior at both molecular and supramolecular levels. The aim of the present work is to systematize current concepts describing how the chemical structure of LPS across different levels of organization, i.e., from individual molecules to supramolecular assemblies, determines their essential physicochemical characteristics, which in turn underlie the wide spectrum of biological functions of these indispensable components of the bacterial cell. We consider various levels of modeling starting from the molecular models up to supramolecular structures such as LPS bilayers and polymer brushes. Treating these models, we focus on relationships between parameters considered at each level of modeling. The first part of the review examines the chemical architecture of LPSs and the sources of their structural variability (Section 2). The next part focuses on the fundamental physicochemical properties of LPS molecules (Section 3), while Section 4 primarily addresses modeling of LPS in solutions. Section 5 and Section 6 treat supramolecular aspects of LPS bilayers, formation of polymer brushes, and colloidal models applied to LPS studies.

## 2. Organization of LPSs

**Figure 1:**
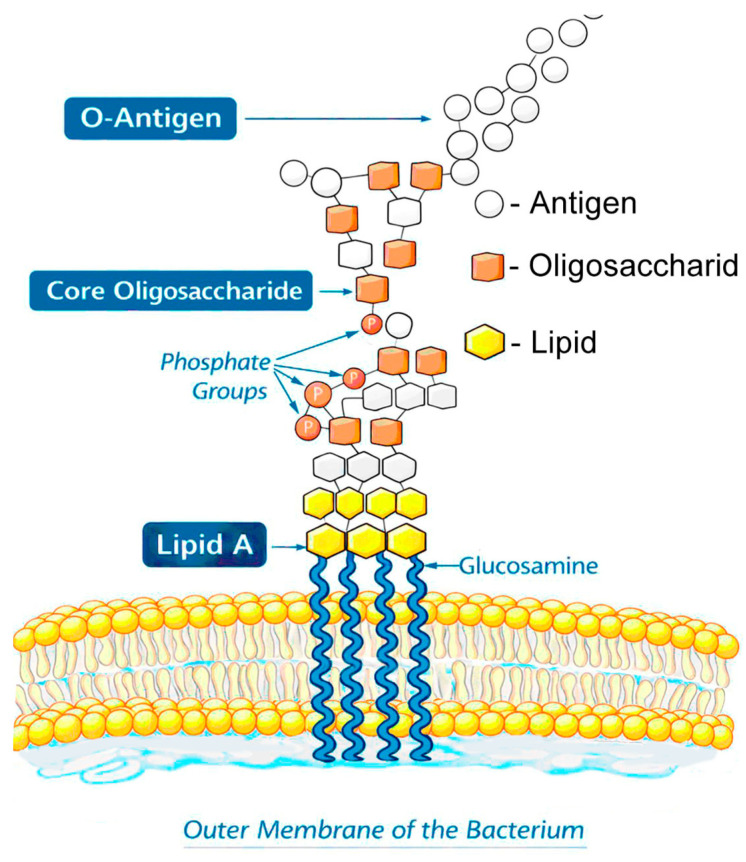
The schematic structure of LPSs embedded in the outer membrane of a Gram-negative bacterium.

Lipopolysaccharides constitute the major components of the outer membrane of Gram-negative bacteria. They perform a wide range of functions, from ensuring the structural stability of the bacterial envelope to modulating the host immune response and mediating bacterial adhesion to various surfaces. The structural organization of LPS is characterized by a high degree of modularity and variability, which is directly reflected in their biophysical and biological properties [5,6]. A common structural feature shared by LPSs from different Gram-negative species is the presence of lipid A and a polysaccharide “headgroup” composed of an oligosaccharide core and an O-antigen. Figure 1 shows a structural sketch of LPSs embedded in the outer membrane.

Lipid A consists of a β-(1→6)-linked D-glucosamine (GlcN) disaccharide phosphorylated at the 1 and 4′ positions, to which four to six fatty acid chains of varying length and saturation are covalently attached. The hydrophobic acyl chains of lipid A are embedded in the outer leaflet of the bacterial membrane, while the phosphate groups confer the negative charge of LPS [1]. The oligosaccharide core connects lipid A to the O-antigen and contains conserved “bridging” residues common to most Gram-negative bacteria. This relatively short polysaccharide segment contributes to membrane rigidity and serves as a binding site for divalent cations (Mg^2+^ and Ca^2+^), thereby stabilizing the charged bilayer. The core region typically comprises 2-keto-3-deoxyoctulosonic acid (Kdo), a non-glycosidic acidic sugar directly linked to the GlcN disaccharide; L-glycero-D-manno-heptoses (Hep), usually present as two consecutive residues that stabilize the linkage between Kdo and outer sugars; and hexoses such as galactose (Gal) and/or glucose (Glc), which form the outer core and vary between species.

The O-antigen is the most variable and externally exposed part of LPS. Typical monomeric units involved in serotyping include N-acetylgalactosamine (GalNAc), galactose (Gal), mannose (Man), and N-acetylglucosamine (GlcNAc) [7,8]. The O-antigen generally consists of dozens of repeating oligosaccharide units that differ in composition, stereochemistry, and glycosidic linkages. Owing to this variability, the O-antigen serves as a primary target for immune antibodies and bacteriophages, while simultaneously protecting bacteria from phagocytosis, enabling immune evasion, and providing a steric barrier. The characteristic combination of GlcN in lipid A, Kdo-Hep-Gal-Glc residues in the core region, and GalNAc-Gal-Man-GlcNAc units in the O-antigen determines the unique physicochemical properties of LPS, including amphiphilicity, charge, conformational flexibility, and serological variability [2]. Depending on the length of the O-antigen, the molecular weight of LPS can vary significantly—from approximately 2.5 kDa for molecules containing only a short oligosaccharide core and lacking an O-antigen to 70 kDa or more for strains expressing long, polymerized O-antigen chains.

O-antigens differ markedly in length, composition, and charge, forming the basis for the serological classification of Gram-negative bacterial strains, including *Pseudomonas aeruginosa*. Most *P. aeruginosa* strains express two distinct O-antigen forms: the common polysaccharide antigen A-band, which is a homogeneous homopolymer of D-rhamnose composed of repeating α-D-rhamnose trisaccharide units (→3)-α-D-Rha-(1→2)-α-D-Rha-(1→3)-α-D-Rha-(1→), and the O-specific B-band antigen. The A-band provides a basal level of immune recognition but exhibits lower antigenic specificity compared to the B-band. In contrast, the B-band is a heteropolymer composed of repeating units containing three to five different sugars, including rare deoxy sugars and uronic acids. Variations in the B-band structure determine the serotype of a given strain according to the International Antigenic Typing Scheme (IATS) [9]. Each serogroup is characterized by a unique repeating oligosaccharide structure (e.g., O5 versus O6).

The A-band homopolymer of D-rhamnose carries fewer negatively charged groups than the B-band, which may influence electrostatic interactions between bacterial cells and surfaces. The A-band is also typically shorter and more rigid, whereas the B-band can be substantially longer, providing enhanced steric protection. Removal of the B-band from *P. aeruginosa* strains has been shown to reduce adhesion forces to silicone surfaces, while the A-band retains basic protective functions but does not confer the same degree of attachment.

The influence of LPS chemical variability on physical parameters was systematically investigated in Ref. [10] by comparing the “smooth” strain PAO1 (A^+^B^+^) with semi-rough mutants such as AK1401 (A^+^B^−^). While PAO1 expresses both O-antigen forms and exhibits maximal adhesion and immunostimulatory activity, AK1401 lacks the B-band antigen. This mutant displays a more pronounced negative surface charge and reduced hydrophilicity, with adhesion force distributions concentrated over shorter separation distances in atomic force microscopy (AFM) measurements. AFM studies further demonstrated that removal of the B-band in AK1401 leads to a decrease in the characteristic range of repulsive interactions and reduced adhesion forces compared to PAO1 [10], highlighting the crucial role of the B-antigen in establishing steric barriers and mediating electrostatic interactions with surfaces. Thus, the serological variability of the O-antigen directly translates into molecular-scale parameters of LPSs, linking chemical diversity to functional roles in adhesion, pathogenicity, and immune response.

## 3. Molecular Properties of LPSs

Lipopolysaccharides are amphiphilic molecules in which the hydrophobic moiety (lipid A) is embedded within the lipid bilayer and preferentially associates with hydrophobic surfaces, while the polysaccharide “headgroup” (core oligosaccharide plus O-antigen) extends into the aqueous phase. Upon contact with biological membranes or solid hydrophobic substrates, the acyl chains of lipid A insert into the bilayer or anchor to the surface, whereas the polysaccharide region remains exposed to the water–interface boundary, maximizing hydration. This amphiphilic nature enables LPSs to simultaneously stabilize the bacterial outer membrane and mediate effective adhesion to a wide variety of surfaces [11,12].

Lipopolysaccharides are characterized by a pronounced negative charge, which originates primarily from phosphate groups in lipid A and carboxylate groups within the core oligosaccharide. This charge governs electrostatic interactions with ions, cellular membranes, and abiotic surfaces. Each LPS molecule typically carries two to four phosphate groups on a lipid A disaccharide (PO_4_^2−^), while additional carboxyl groups are contributed by 3-deoxy-D-manno-oct-2-ulosonic acid (KDO) and certain heptose residues in the core region [13]. As a result, the overall formal charge of a single rough-type LPS molecule can reach −6e (excluding counterions) and is determined directly by the chemical structure of the polymer [3,14]. The typical values of the charge as well as other key molecular characteristics of LPSs are listed in Table 1.

LPS molecules exhibit significant conformational flexibility, particularly within the O-antigen region, which strongly influences their behavior in solution, interactions with surfaces, and involvement in biological processes. The contour length $L_{c}$ of an LPS depends on the number of repeating units in the O-antigen. For *P. aeruginosa* strain PA103, the average LPS length is approximately 35 nm, whereas mutants with elongated O-chains exhibit lengths of 100–200 nm, and wild-type strains can reach up to 280 nm [15]. As noted in Section 2, the contour length varies depending on the serotype. For example, studies show that for serotype O5, the length is about of 40–65 nm for fully extended molecules [5].

The persistence length $L_{p}$ characterizes the stiffness of the polymer chain and depends sensitively on both molecular structure and ionic conditions in solution. The behavior of LPS can be described within the framework of the semiflexible (worm-like chain, WLC) model [16], in which the persistence length is related to the bending rigidity $B_{s}$ and temperature *T* as follows:

(1) $$L_{p}=B_{s}/k_{B}T$$

where $k_{B}$ is the Boltzmann constant. Importantly, the flexibility of LPSs is highly sensitive to ionic strength and pH. At low ionic strength and high pH, the negative charges of phosphate and carboxyl groups enhance intrachain electrostatic repulsion, leading to increased chain stiffness. In contrast, the presence of divalent cations such as Mg^2+^ or Ca^2+^ effectively screens these charges, reducing $L_{p}$ and increasing chain flexibility. A decrease in the persistence length of LPSs with increasing ionic strength (e.g., upon addition of KCl) has been experimentally confirmed by atomic force microscopy (AFM) measurements [10].

**Table 1:** Key molecular characteristics of LPS.

Parameter	Description	Range/Value	Main Factors Affecting the Parameter
Molecular weight	Mass of a single LPS molecule	2.5–70+ kDa [3,17]	Length and composition of O-antigen
Contour length $L_{c}$	End-to-end contour length of the molecule	~35–280+ nm [15]	Number of O-antigen repeating units
Formal charge	Total negative charge of the molecule	−4 to −6 e [14]	Number of phosphate and carboxylate groups
Amphiphilicity	Balance between hydrophobic and hydrophilic domains	High (lipid A + polysaccharide headgroup) [1,3]	Chemical structure
Conformational flexibility	Ability to undergo spatial conformational changes	Depends on ($L_{p}$) and ionic strength [18]	Chain length, charge density, ions
Persistence			
length $L_{p}$	Polymer chain stiffness	Estimated using the WLC model [19,20]	Charge density, ionic strength, molecular structure

The persistence length $L_{p}$ of a polymer chain is commonly expressed [18] as the sum of intrinsic stiffness $L_{o}$ and electrostatic contribution $L_{el}$:

(2) $$L_{p}\;={\;L}_{o}+L_{el}.$$

To estimate these contributions, the Odijk–Skolnick–Fixman (OSF) model is widely employed [19,20]. For lipopolysaccharides, which carry a high density of negative charges, the OSF framework provides a useful means to assess the extent to which electrostatic repulsion between phosphate groups and carboxylates increases the persistence length and, consequently, affects conformational flexibility. The OSF theory describes the electrostatic contribution to polymer stiffness and is broadly applied to estimate the effective persistence length of polyelectrolytes. In the case of surface-grafted charged chains, these concepts provide a microscopic justification for the parameters used in polymer brush theories (see Section 5). In LPS, the dominant charged groups are the phosphate moieties of lipid A and the carboxylate groups of KDO and heptose residues. The average spacing between these charged groups is typically estimated to be in the range of 0.5–1 nm. The OSF model is formally valid for strongly charged and relatively stiff polymer chains. For soft or weakly charged chains at low ionic strength, additional effects such as thermal fluctuations and logarithmic corrections must be taken into account [21]. In the regime of weakly charged polymers, the electrostatic blob model (see Section 5) provides a more appropriate description [22].

As can be seen from Table 1, the molecular parameters vary greatly depending on the composition of the solution, the presence of ions, as well as the growth conditions and the serotype and strains of the bacteria. Such variability rather complicates the modeling of LPS in solutions as well as various supramolecular LPS ensembles. The latter may be characterized by certain supramolecular parameters. Relationships between the molecular and supramolecular characteristics are not clear and will be discussed below.

## 4. Lipopolysaccharides in Solutions

**Figure 2:**
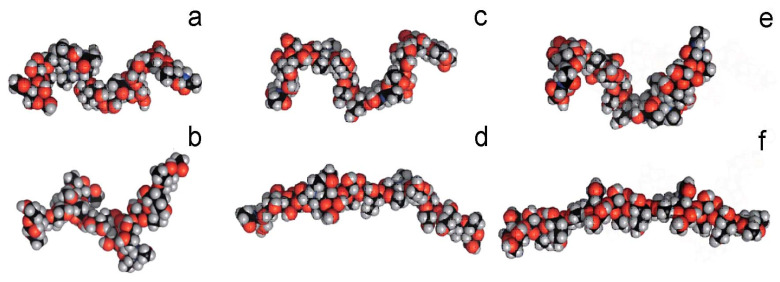
Various conformations of O-antigen LPS of *E.coli* O5ac (**a**,**b**) and O5ab (**c**–**f**) conformations of the O-antigen PS of *E. coli*, obtained by conformational search with the use of NMR data [32].

In molecular dynamics studies of carbohydrate biopolymers in solutions, complete LPS assemblies are rarely modeled in their entirety. Instead, simulations typically focus on individual polysaccharide fragments or representative chain segments such as lipid A [23,24] or antigen [25,26,27]. This approach is dictated both by computational limitations and by the structural complexity of LPS. Nevertheless, even in such a simplified form, polysaccharides remain challenging objects for molecular modeling. Experimental studies demonstrate that polysaccharide chains can adopt a wide range of conformational states, including relatively rigid extended conformations, single- or triple-helical structures, as well as flexible statistical coils and aggregated assemblies. Figure 2 demonstrates typical conformations of O-antigen PS of *Escherichia coli*, obtained by conformational search with the use of NMR data [17]. Numerous simulations have confirmed this statement [25,26,27]. Moreover, MD simulations of Re-LPS (rough mutant LPS, i.e., truncated LPS form) have indicated that stability of O-chain conformations is strongly affected by the presence of ions [28,29,30].

In particular, for many bioactive polysaccharides, the formation of a triple helix is considered a structurally and functionally relevant state, whereas, under different conditions, the same chains may transition into disordered coil-like configurations [31]. This conformational diversity is closely linked to both the chemical nature of polysaccharides—such as the type of monomeric units, glycosidic linkages, degree of branching, and presence of functional groups—and to environmental parameters, including ionic strength, pH, solvent type, and the presence of multivalent cations.

Experimental evidence indicates an exceptional sensitivity of polysaccharide chain conformations to these factors, which substantially complicates the development of universal models for polysaccharides in solutions. Additional complexity arises from the intrinsic tendency of polysaccharides to aggregate and to form dynamic supramolecular structures as well as from other properties discussed above. These effects are particularly pronounced for extracellular polymeric substances (EPSs) of microbial origin [30,31].

Within atomistic simulations, classical force fields such as GLYCAM and CHARMM are widely employed to describe polysaccharides in solutions [33,34], a comprehensive overview of available force fields and their limitations is provided in Ref. [22]. Despite significant progress in force field development, numerous studies point to fundamental limitations of these models when applied to carbohydrate systems. One of the key issues is the inadequate reproduction of experimentally observed conformational states of polysaccharides in solution. In particular, several molecular dynamics studies report a pronounced tendency toward chain collapse and the formation of compact coil-like structures [23], which are not directly supported by experimental observations. Despite the chemical variety of LPS, lipid A for various LPS structures reveals common conformational and packing features [35,36].

Analysis of the underlying mechanisms of such collapse indicates that it arises from the cumulative effect of intramolecular interactions, which are primarily electrostatic in nature. Existing force fields often exhibit excessive chain aggregation even at low concentrations as well as an unsatisfactory description of the thermodynamic and structural properties of polysaccharide solutions [37]. To mitigate these artifacts, several authors have proposed modifications of the partial charges assigned to carbohydrate groups [38]. Taken together, these findings indicate that modeling polysaccharides in solution remains a methodologically challenging task. While modern force fields can provide qualitative insight into possible conformational states, their predictive power with respect to specific structural motifs remains limited. Achieving quantitative agreement with experimental data often requires the use of specialized charge schemes or the adoption of more sophisticated interaction models.

From an experimental perspective, the behavior of LPS in solution is highly diverse due to its pronounced amphiphilic character. Upon increasing the LPS concentration, a critical aggregation concentration (CMC) is reached, above which individual LPS molecules associate into aggregates in order to minimize the free energy of the system. The nature of LPS assemblies strongly depends on whether the concentration is below or above this threshold. Below the CMC, LPS molecules are predominantly dispersed as monomers or small associates. Above the CMC, LPS molecules form micellar structures—typically spherical aggregates with diameters in the range of 5–20 nm—where the hydrophobic lipid A chains are sequestered in the interior, while the polysaccharide chains extend into the surrounding aqueous phase.

In addition to micelles, larger LPS aggregates have also been observed. Transmission electron microscopy and dynamic light scattering experiments have revealed the formation of non-membranous spherical particles with diameters of up to ~200 nm, often referred to as LPS vesicles, which mimic certain features of the bacterial outer membrane [39]. The critical aggregation concentration of lipid A and LPS has been determined using fluorescence probe techniques [40]. For lipid A, the CMC was found to lie in the range of 10–20 μg/mL, while the presence of polysaccharide chains reduces this value due to increased hydrophilicity and additional intermolecular interactions. Typically, critical self-assembly concentration is determined from surface tension isotherms or from changes in fluorescence intensity as a function of LPS concentration. Light scattering measurements indicate that above the CMC, the average radius of gyration of LPS micelles ($R_{g}$) is approximately 105 nm, while the hydrodynamic radius ($R_{h}$) is about 95 nm, yielding a shape factor ρ = /$R_{h}$ ≈ 0.86, consistent with nearly spherical aggregates [41]. Below the CMC, pre-micellar oligomers are observed with $R_{g}$ ≈ 56 nm and $R_{h}$ ≈ 60 (ρ ≈ 0.93), corresponding to more diffused yet still relatively large supramolecular assemblies. LPS vesicles (also referred to as outer membrane vesicle-like structures) are composed of a double bilayer of LPS and phospholipids and can reach diameters of 100–200 nm, depending on concentration and ionic strength [39,42].

One of the main objectives of LPS studies in solution is to identify the relationship between LPS conformation and their biological activity. MD simulations and various physicochemical studies confirm that this relationship is clearly evident [43,44]. LPS activity is linked to the conformation of lipid A, which is controlled by a large number of molecular parameters (see Section 3) and the ability to form various 3D structures in solution. The main molecular parameter determining both agonistic and antagonistic effects is the charge of lipid A [45]. As concluded in [25], the size as well as shape of micelles is governed by the charge and positions of lipid A in LPS structure, while the latter depends strongly on the growth conditions and presence of ions.

## 5. Lipopolysaccharide Bilayers

The outer membrane (OM) of Gram-negative bacteria represents a unique asymmetric lipid bilayer that performs critical functions in cellular protection and selective permeability. The OM consists of two distinct leaflets: an inner and an outer layer. The inner leaflet is predominantly composed of phospholipids similar to those found in the inner bacterial membrane, whereas the outer leaflet is mainly formed by lipopolysaccharides. The presence of LPSs confers distinctive properties to the membrane, including a high resistance to hydrophobic molecules and antimicrobial agents. This asymmetry is essential for the barrier function of the OM, effectively preventing the penetration of toxic compounds and antibiotics. In addition, the OM contains porins—protein channels that enable passive transport of hydrophilic molecules such as ions and small sugars. The stability of the OM is further enhanced by divalent cations, such as Mg^2+^ and Ca^2+^, which screen the negative charges of LPS phosphate groups, promote tight molecular packing, and increase membrane robustness. Thus, the architecture of the outer membrane of Gram-negative bacteria constitutes a highly organized and complex structure that ensures cellular protection and participates in a wide range of biological processes [46].

LPS bilayers represent one of the most extensively studied systems in both experimental and computational investigations of lipopolysaccharides. Although the first modeling attempts date back to the mid-1990s, systematic simulations of LPS bilayers have become widespread only over the past 15 years, largely due to the development of coarse-grained molecular dynamics (CG MD) approaches. Fully atomistic simulations of LPS bilayers are computationally demanding, as they typically require systems containing more than one million atoms. The use of CG MD reduces the number of particles by at least an order of magnitude, thereby decreasing computational time by several orders of magnitude. Nevertheless, accurate modeling of LPS bilayers that include O-antigen chains remains challenging, since the conformational relaxation of antigenic polysaccharides is extremely slow and requires very long simulation times to reach equilibrium.

**Figure 3:**
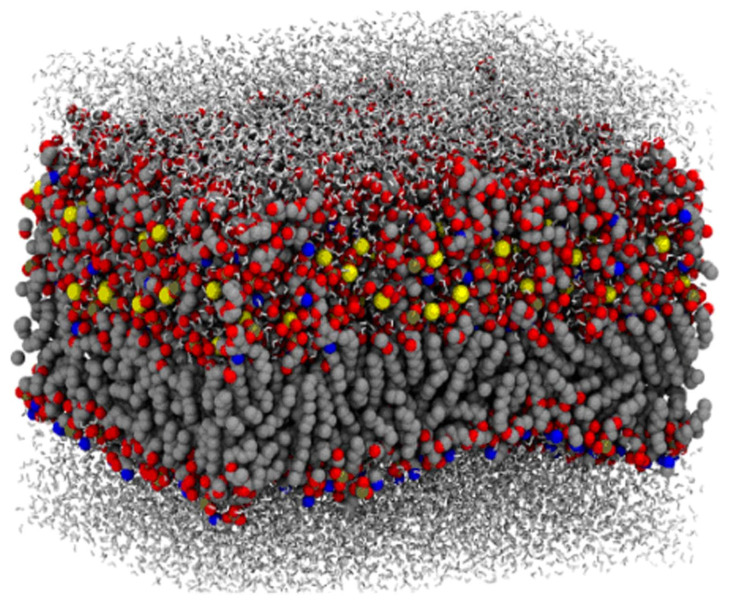
Molecular representation of the LPS bilayer. The bilayer atoms are spheres (gray—carbon, red—oxygen, blue—nitrogen, yellow—Ca^2+^), and water molecules are sticks (white) [52].

As a consequence, most simulation studies focus either on Ra LPS bilayers (rough chemotype lacking the O-antigen polysaccharide) or on Re LPS systems (deep rough chemotype lacking both the O-antigen and most of the outer core oligosaccharide). The primary goals of such simulations typically include determining membrane architecture (see, e.g., [47,48]), calculating electrostatic potential profiles [49], analyzing the spatial distributions of lipids, ions, and water molecules, and estimating the thickness of the hydration layer [14,50,51], as well as examining how these properties vary with LPS chemical composition. Figure 3 shows a molecular representation of the LPS bilayer obtained by CG MD [52].

Accurate parameterization of force field is required to reproduce reliable OM architecture. The field parameterization is to be based on the molecular properties of LPS, and it reflects the experimentally observed LPS behavior in solutions. For example, to develop a realistic CG model of the OM bilayer, the authors of [53] take into account experimental data on phase transitions, their temperatures and ordering parameters of LPS in the OM bilayer. On the other hand, the simulated architecture depends sufficiently on the choice of partial charges. Early MD models have applied CNDO calculations of these charges [54], whereas the current CG models are able to reproduce electrostatic potentials and bilayer architecture with the use of parameterized charges [55,56].

Supramolecular structure of LPS bilayers is reflected in various physicochemical parameters such as hydrophobic thickness, area per lipid, tilt angles, hydrodynamic radius, etc. Below, we outline the most significant parameters. The hydrophobic thickness of the lipid bilayer is defined as the distance between the centers of mass of the hydrophobic tails in opposing leaflets of the membrane. This parameter depends on the length and degree of saturation of the fatty acid chains in lipid A and typically falls within the range of 2–5 nm. Longer and more saturated acyl chains increase bilayer thickness, thereby enhancing the barrier function of the membrane. For example, Ref. [57] demonstrated that increasing the acyl chain length from 14 to 26 carbon atoms leads to an increase in hydrophobic thickness and a concomitant decrease in membrane permeability to small molecules. Hydrophobic thickness plays a crucial role in maintaining membrane structural integrity and mediating interactions with membrane proteins. A mismatch between membrane thickness and the length of hydrophobic segments in proteins may result in membrane deformation or protein conformational changes, ultimately affecting protein function.

Another important structural parameter is the area per lipid (APL), which reflects the packing density of lipid molecules within the membrane. For pure LPS bilayers, atomistic simulations typically report APL values in the range of approximately 1–2 nm^2^ [58]. The presence of long O-antigen chains can lead to an increase in APL, as additional lateral space is required to accommodate bulky polysaccharide side chains. Structural features of LPS, including the presence of O-antigens and variations in lipid A composition, significantly influence APL values. For instance, simulations of the outer membrane of *P. aeruginosa* have shown that APL varies depending on the specific LPS components present and their interactions with the surrounding environment [58].

Moreover, the presence of different lipid species and their relative proportions can alter membrane packing density. Increasing temperature typically leads to higher APL values due to enhanced thermal fluctuations, whereas the presence of ions, particularly divalent cations, can stabilize the membrane and reduce APL. Variations in APL influence membrane permeability, interactions with proteins and other biomolecules, and bacterial resistance to external stresses. Consequently, accurate determination of APL and a detailed understanding of the factors affecting it are essential for elucidating bacterial physiology and for developing antimicrobial strategies. Overall, APL serves as an important indicator of the structural organization and functional state of lipopolysaccharide bilayers, reflecting their adaptation to changing environmental conditions.

Recently, the development of improved force field parameters for O-antigen polysaccharides has enabled active investigations of full (smooth) LPS bilayers that explicitly include antigenic chains. In addition to APL and $R_{g}$, these studies also employ the average tilt angle (Θ) as a key descriptor [51]. Molecular dynamics simulations demonstrate that the thickness of the antigenic layer increases linearly with the number of repeating antigen units, while the tilt angle decreases monotonically [51]. These observations indicate that the antigenic component of LPS can be effectively described within the framework of worm-like chain models.

MD modeling focuses on the reproduction of supramolecular parameters. For example, the CG model based on MARTINI force field accurately reproduces APL and bilayer thickness [59], whereas models based on CHRAMM [35] and GLYCAM [58] force fields yield realistic surface pressure, distribution of charge and mass densities and can provide an accurate account of the ion influence of supramolecular parameters. Another way of CG modeling is based on comparison with biophysical experiments. The authors of [36] combine MD simulations and NMR data to reconstruct LPS conformations, while authors of [60] use a combination of MD simulations and analysis of reflectivity data. The MD models are often used to validate biophysical experiments of protein–membrane interactions (see, for example, [61,62,63]).

Although the general architecture of LPS is conserved among Gram-negative bacteria, substantial structural variability exists in the lipid A acylation pattern, core oligosaccharide composition, and O-antigen length and chemistry. Smooth and rough chemotypes, core truncations, and differences in O-antigen repeat units directly affect grafting density, brush thickness, surface charge distribution, and membrane packing density. These structural variations translate into measurable differences in steric repulsion, electrostatic screening, and permeability barriers, ultimately influencing bacterial resistance to antimicrobial peptides and antibiotics. Therefore, species-specific LPS architecture should be considered an important determinant of supramolecular membrane mechanics and interfacial interactions.

**Figure 4:**
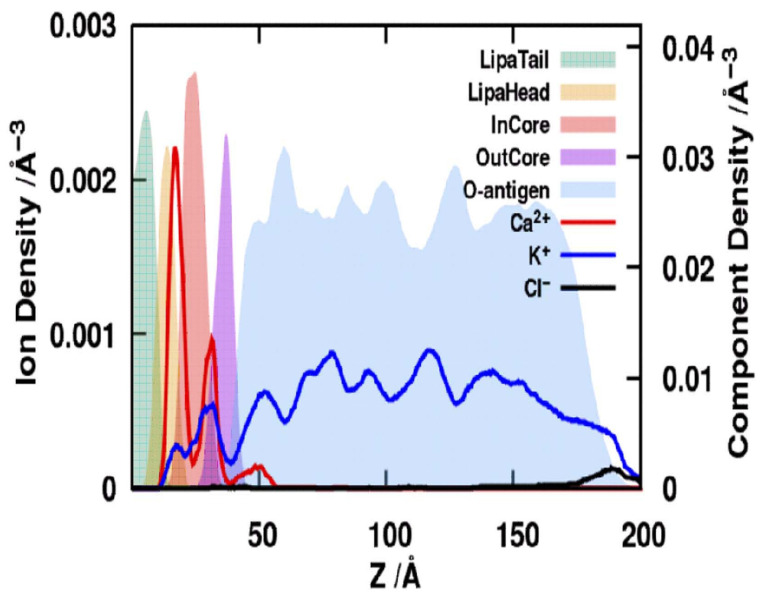
Distributions of ions and LPS components along the Z-axis in the *E. coli* O91 LPS10 bilayers obtained by combination of NMR and CG MD studies [51].

Beyond these pronounced biophysical effects, CG MD simulations allow a systematic exploration of the influence of a wide range of physicochemical parameters, including ion composition and concentration, lipid and antigen composition, and interactions with various membrane proteins [48,58,64]. Figure 4 demonstrates a typical distribution of water molecules, ions, and LPS counterparts in coarse-grained simulated *E. coli* bilayers [51]. Table 2 lists all the discussed key properties of supramolecular LPS-based structures, including bilayers, polymer brushes, and colloidal models.

## 6. Polymer and Colloidal Models of LPS

**Figure 5:**
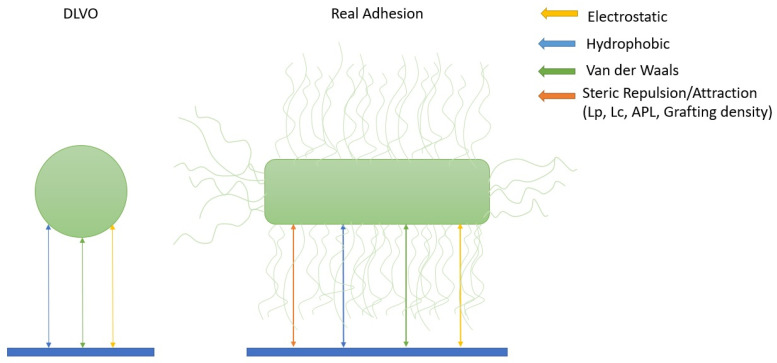
Conceptual comparison of the classical DLVO model and real bacterial adhesion mediated by LPS layers.

Although the models discussed above allow one to resolve, in numerical experiments, the influence of molecular details on the architecture and function of biomembranes, identifying these effects directly in many biophysical experiments remains extremely challenging. For this reason, highly coarse-grained polymer and colloidal models are widely employed to interpret experimental data. Some of the molecular parameters of LPS such as persistence length, APL, etc., are used to describe colloidal and polymer models; however, new supramolecular characteristics are required for ultra-coarse-grained modeling. The most primitive colloidal descriptions are based on the extended DLVO theory (XDLVO) [69,70] (See Figure 5), in which LPS are reduced to an effective surface layer interacting via Coulombic, van der Waals, and hydrophobic forces.

First, we start with the consideration of colloidal models. In the classical DLVO model (left), adhesion is approximated by a balance of electrostatic and van der Waals interactions (and hydrophobic effects in xDLVO) between a rigid particle and a surface. In contrast, real-world adhesion of Gram-negative bacteria (right) is strongly dependent on the presence of a soft, permeable lipopolysaccharide (LPS) layer, where additional factors such as steric repulsion/attraction, hydrophobic effects, and polymer chain properties (e.g., persistence length $L_{p}$, contour length $L_{c}$, area per lipid (APL), and grafting density) determine the interaction potential and force profiles.

In practical studies of bacterial adhesion and interfacial interactions, the complex properties of the bacterial surface are, thus, mapped onto a set of effective parameters accessible to experimental determination. These include the contact angle, which is used to estimate surface energy and the relative contributions of hydrophobic and polar interactions, as well as the ζ-potential, which reflects the electrokinetic properties of the “bacterial surface–solution” interface. Despite their integral and condition-dependent nature, these quantities are widely used for phenomenological descriptions of bacterial adhesion and for comparison of experimental results obtained by different techniques (see details in [71,72]).

Under native conditions, the ζ-potential of bacteria typically lies in the range −30 to −60 mV [73], although this range varies between strains; more negative values generally indicate higher electrostatic stability and resistance to aggregation. Upon lowering the pH to 3–5, partial protonation of phosphate groups occurs, reducing the net negative charge and shifting the ζ-potential toward zero (down to approximately −10 mV) [74,75]. At physiological pH (7–8), LPS are fully dissociated and carry their maximum negative charge [42]. The average ζ-potential of *E. coli* is approximately −44.2 mV, whereas, for Staphylococcus aureus, it is about −35.6 mV. These values depend on strain, serotype, and environmental conditions and reflect the higher surface charge density of *E. coli* due to the presence of LPS in the outer membrane. Importantly, the bacterial ζ-potential is sensitive to multiple factors, including pH, ionic strength, and the presence of cationic agents [76]. For example, treatment of *E. coli* with cationic compounds such as cetyltrimethylammonium bromide (CTAB) or polymyxin B leads to a reduction in the negative ζ-potential, correlating with increased membrane permeability and, in some cases, cell death [65]. On the other hand, a simple approach based on evaluations of ζ-potential cannot explain the variety of bacterial adhesion caused by LPS [77]. Moreover, for sophisticated models, coupled electrostatic and mechanical properties of bacterial interfaces in aqueous media are required [78]. The models are designed to account for volume charge distributions as well as heterogeneity of surface LPS [79].

Bacteria coated with a dense layer of lipopolysaccharides can, to a good approximation, be considered soft particles, in which ions from the surrounding solution freely penetrate the polysaccharide “brush” layer. Within this framework, the electrostatic properties of the interface are determined not only by the surface charge but also by the spatial distribution of fixed charges inside the polymer layer. This naturally leads to models in which the LPS layer is treated as a volume-charged, permeable medium and the electrostatic potential is calculated while explicitly accounting for ion penetration into the layer [80,81].

The soft particle model developed by Ohshima and co-workers [82,83] incorporates the distribution of fixed charges within the LPS layer, the high permeability of the polymer brush to ions, and a shift in the so-called slipping plane to a depth on the order of λ. This parameter characterizes the effective “softness” of the particle and represents the ratio of the solvent viscosity to the friction coefficient of the polymer chains. It should be emphasized, however, that the soft particle model relies on several simplifying assumptions. In particular, the polymer layer is treated as a continuous, spatially homogeneous, and stationary medium, whereas real bacterial envelopes exhibit pronounced structural heterogeneity, polydispersity, and dynamic mobility of LPS chains. Furthermore, the model neglects possible conformational rearrangements of the polymer layer upon particle approach or under externally applied forces, which may be important for interpreting AFM experiments [84,85,86]. In addition, ion distributions within the polymer layer are described within a mean-field approximation, precluding explicit consideration of specific ion effects, correlations, and localized counterion binding to LPS functional groups.

As a result, ζ-potentials calculated using soft particle theory often differ substantially from classical estimates, frequently exhibiting larger absolute values and showing improved correlation with experimental data on electrophoretic mobility and bacterial adhesion [87]. Incorporation of this “soft” potential into DLVO-based calculations enables more accurate predictions of interfacial interactions involving bacteria [88,89]. In contrast, classical electrical double-layer models [90] severely underestimate the role of the polymer layer and electrostatic screening. The soft particle model yields a potential profile that decays gradually over hundreds of nanometers, in agreement with AFM force–distance profiles measured for *E. coli* and *Pseudomonas* species [10]. Accounting for the “softness” of the LPS layer, therefore, not only resolves discrepancies between theory and experiment but also provides insight into why significant repulsive interactions persist over tens of nanometers even under strong screening by divalent cations (Mg^2+^, Ca^2+^), and how variations in polysaccharide chain length or environmental conditions (pH, ionic strength) modulate electrostatic adhesion forces. Relevance of the soft approach to bacterial adhesion has been intensively studied [91,92].

Numerous experimental measurements have indicated that neither the DLVO nor the XDLVO approach are able to accurately quantify bacterial adhesion; hence, an account of non-DLVO interactions are required [93,94]. Although the origin of these interactions may not be directly related to LPS, the latter significantly affects the bacterial attachment. Moreover, the LPS effect may increase and decrease the attachment simultaneously; the balance depends on the molecular details described above, such as persistence length $L_{p}$, volume fractions of antigens and other molecular characteristics of LPS [95]. For example, the authors of [94] have argued that the adsorption energy decreases due to polymer repulsion caused by LPS. On the other hand, the authors of [93] found evidence in favor of steric attraction, which inhibits electrostatic repulsion. They found that the presence of anionic LPS increases the bacterial attachment to surface with respect to non-polysaccharide macromolecules such as lipids and proteins.

**Figure 6:**
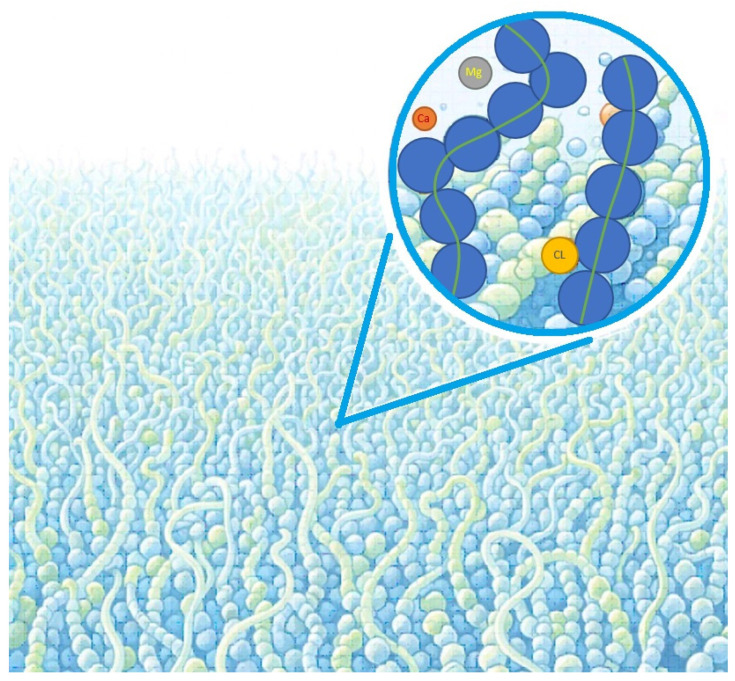
Schematic model of polymer brushes and blobs (see explanation in the text).

**Figure 7:**
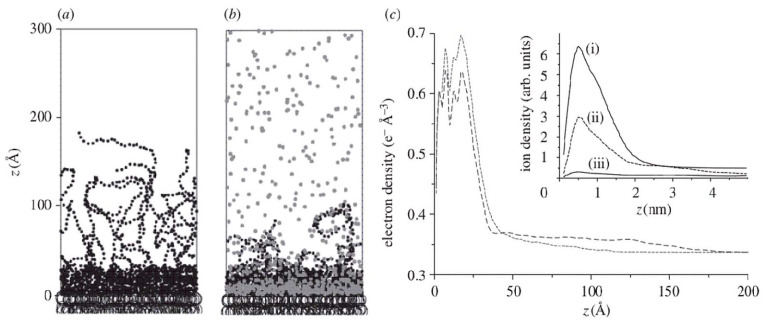
Simulations in the absence (**a**) and presence (**b**) of Ca^2+^ hydrocarbon moieties are indicated by open large circles, saccharide groups by small, filled black circles and Ca^2+^ ions by small, filled gray circles. (**c**) Time-averaged electron density profiles calculated from the simulations in the absence (broken line) and presence (dashed line) of Ca^2+^. The inset shows distributions of: (i) K^+^ in the absence of Ca^2+^, (ii) Ca^2+^, (iii) K^+^ in the presence of Ca^2+^ [98].

In polymer-based models, the supramolecular organization of the LPS layer is commonly described as a polymer brush [96,97]. This approach, conceptually analogous to classical polymer brush theories [67,68], allows both qualitative and quantitative interpretation of force–distance profiles obtained by atomic force microscopy (AFM) [90] as well as the prediction of the dependence of steric repulsion—and, in some cases, attraction—on the length and grafting density of polymer chains. Here, the grafting density refers to the number of O-antigen polysaccharide chains attached to the membrane per unit surface area. At high grafting densities, neighboring polymer chains experience strong interchain repulsion, which leads to brush stretching, additional compaction of the layer, and an increase in the characteristic interaction length scale. The key parameters of such a polymer brush are the effective brush thickness L and the surface grafting density Γ. In general, detailed modeling of the structure of polymer brushes is rather complicated due to various scales of interaction forces. A blob concept [67,68] assumes the behavior of polymer brushes to be mainly controlled by blob–blob interactions. This approach treats blob size as a length scale below which the polymer chain may be considered to be unperturbed by other forces [67]. Figure 6 demonstrates a sketch of the blob concept applied to the LPS layer, where blobs are indicated by dark blue spheres. Another example of coarse-grained modeling is considered in Ref. [98], where a minimal CG model of LPS molecules has been constructed to account for electrostatic characteristics of O-antigen. The model has been applied to treat the X-ray reflectivity data for LPS bilayers [97,99,100]. Even the minimal model explains the influence of solvent composition, namely, ion concentration on the properties of polymer brush (see Figure 7).

In many modeling studies, the grafting density is often assumed to be equal to the density of lipid molecules in the bilayer (i.e., the inverse of the area per lipid, APL). However, experimental data obtained by AFM [66] and neutron reflectometry [101] indicate that less than 10% of LPS molecules actually carry an O-antigen component (see Table below). This finding highlights the intrinsic heterogeneity of the LPS layer and suggests that simplified assumptions regarding uniform grafting may lead to significant overestimation of brush density.

There is currently no consensus regarding the dominant nature of interactions between polymer brushes formed by O-antigens and external molecules. Some studies treat the antigenic polymer brush as effectively uncharged, reducing its interaction with proteins, primarily due to steric constraints [102]. Other works, in contrast, argue that electrostatic interactions between peptides and the charged polymer brush play a decisive role in governing adhesion and binding phenomena [103,104]. AFM measurements further demonstrate that the thickness of the polymer layer strongly depends on both pH and ionic strength [66], providing direct evidence for the substantial contribution of electrostatic interactions to the structure and mechanics of the LPS brush.

The parameters listed in Table 3 correspond to the colloidal–polymer description of LPS-covered bacterial surfaces used to interpret AFM force measurements and adhesion behavior. The bulk ionic strength (ρq, mM) determines electrostatic screening in the surrounding electrolyte. The effective ζ-potential (−ζ, mV) represents the electrokinetic potential of the soft interface. The parameter λ (nm) characterizes the hydrodynamic softness and permeability of the LPS layer within the soft particle framework rather than its geometrical thickness. The Hamaker-related parameter A reflects the magnitude of van der Waals interactions contributing to the overall force balance. Within the polymer brush model, the brush thickness (L, nm) and grafting density (Γ, nm^−2^) describe the structural organization of O-antigen chains extending from the membrane surface. Together, these parameters provide a physically consistent description of electrostatic, steric, and dispersion contributions governing bacterial adhesion beyond the classical DLVO approximation.

When an AFM probe approaches an LPS membrane, an extended repulsive interaction zone spanning hundreds of nanometers is typically observed, corresponding to the stretching of polysaccharide brushes formed by O-antigens and extracellular polymeric components (ECP). This effect was characterized in detail in [108], where measurements on *P. putida* KT2442 revealed that the range of repulsion varied with ionic strength and biopolymer composition. A key advantage of the polymer brush model for LPS is its ability to directly relate O-antigen morphology (chain length, size, and grafting density) to AFM force profiles, as well as to explain why pronounced steric repulsion persists over hundreds of nanometers even under strong electrostatic screening by Mg^2+^ or Ca^2+^ ions. Importantly, the model also enables predictive assessments of how genetic modifications (e.g., changes in O-antigen length) or environmental parameters (salinity, pH) influence bacterial adhesion properties. Finally, we list in Table 3 typical values of the discussed parameters of LPS bilayers and LPS-coated systems, which were obtained experimentally or estimated within the theoretical studies.

## 7. Conclusions

Although there are strict relationships between the molecular characteristics of LPS and their manifestation in biophysical experiments, the correlation between the supramolecular and molecular properties of LPS is far from trivial and sometimes not obvious, as it is masked by the variability of the chemical structure of LPS in many cases. This creates significant difficulties in the feasibility of a single, universal approach for biophysical modeling of LPS. In this review, we have attempted to identify these relationships to the best of our ability. To summarize, we note the following conclusion. Rather than being merely structural components of the outer membrane, lipopolysaccharides act as dynamic regulators of membrane mechanics, interfacial electrostatics, and steric interactions. The analysis presented in this review indicates that several structural parameters play a decisive role in determining bacterial behavior: O-antigen length and grafting density govern steric repulsion and brush thickness; surface charge density and its ionic screening control electrostatic stability and interaction with antimicrobial peptides; hydrophobic thickness and area per lipid define membrane packing and permeability.

Importantly, species-specific variations in core composition and O-antigen architecture translate into measurable differences in membrane rigidity, ζ-potential, and susceptibility to antibiotics. These findings suggest that future experimental efforts should prioritize quantitative characterization of grafting density, ion-dependent brush elasticity, membrane packing parameters, and electrostatic profiles under physiologically relevant conditions.

By integrating atomistic simulations, coarse-grained models, and colloidal theories within a unified framework, this review provides a multiscale perspective that relates molecular architecture to measurable biophysical parameters. Such integration is essential for rational design of antimicrobial strategies and predictive modeling of bacterial adhesion and membrane stability.

## Figures and Tables

**Table 2:** Key properties of supramolecular LPS-based structures.

Parameter	What Represents	Main Affecting Factors
Area per lipid (APL)	Packing density of lipid molecules in the bilayer	Length and volume of the O-antigen; ionic charge screening by Ca^2+^/Mg^2+^; temperature and pressure [58]
Hydrophobic thickness	Distance between the centers of mass of hydrophobic cores of opposing bilayer leaflets	Length and saturation of lipid A fatty acid chains; degree of cross-linking by multivalent ions [57]
Persistence length ($L_{p}$)	Stiffness of the polysaccharide chain	O-antigen length; polysaccharide charge (number of phosphates/COO^−^ groups); ionic strength and pH (OSF model) [19,20]
Hydrodynamic radius ($R_{h}$)	Effective size of the chain in a moving solvent	Size and flexibility of the O-antigen; salt concentration and buffer composition [41]
Total (formal) charge	Sum of negative functional groups per LPS molecule	Number of phosphate groups (PO_4_^2−^) in lipid A; number of COOH groups in the core (KDO, heptoses); environmental pH [3,14]
ζ-potential	Surface electrostatic potential of the particle	Overall LPS charge; ionic strength and ion composition (monovalent vs. divalent cations); pH; adsorbed peptides [65]
Grafting density	Number of O-antigen anchoring points per unit surface area	Core region composition; serotype; expression of A- vs. B-band O-antigens [66]
Polymer brush stiffness	Strength of steric repulsion between chains	Persistence length ($L_{p}$); grafting density; O-antigen length; temperature; ionic strength [67,68]
Tilt angle (Θ)	Orientation of polymer chains relative to the membrane normal	Chain length; packing density; ionic effects acting on LPS [51]

**Table 3:** Experimentally and theoretically determined parameters of LPS layers and LPS-coated systems.

λ (nm)	ρq (mM)	A (*10^11^ N)	−ζ (mV)	L (nm)	Γ (*10^2^ nm^−2^)	Object/Method	Refs.
1.4	7.5	0.64	28.4	n/a	n/a	*E. coli* cell–nanoparticle interaction, AFM	[105]
1.0	7.2	1.0	17.3	280	0.17	LPS layer, AFM + brush model	[66]
n/a	10	n/a	37	171	n/a	*P. aeruginosa*, AFM	[15]
n/a	45	n/a	5	40	n/a	Bacterial surface, high ionic strength, AFM	[106]
0.3	30	18	28	50	1.2	Soft particle + AFM	[107]
n/a	n/a	n/a	18	n/a	0.15	LPS brush + proteins, NR	[101]

## Data Availability

The original contributions presented in this study are included in the article. Please direct further inquiries to the corresponding author.
